# Levels of vitamin D-associated cytokines distinguish between active and latent tuberculosis following a tuberculosis outbreak

**DOI:** 10.1186/s12879-019-3798-5

**Published:** 2019-02-13

**Authors:** Yoonki Hong, Youngmi Kim, Jae Jun Lee, Myung Goo Lee, Chang Youl Lee, Youlim Kim, Jeongwon Heo, Seon-Sook Han, Seung-Joon Lee, Woo Jin Kim, Ji Young Hong

**Affiliations:** 1 Department of Internal Medicine, Kangwon National University Hospital, School of Medicine,Kangwon National University, Chuncheon, Republic of Korea; 2 Lung Research Institute of Hallym University College of Medicine, Chuncheon, Gangwon-do Republic of Korea; 3 Institute of New frontier Research, Hallym University College of Medicine, Chuncheon, South Korea; 4 Division of Pulmonary, Allergy and Critical Care Medicine, Department of Internal Medicine, Chuncheon Sacred Heart Hospital, Hallym University Medical Center, 77, Sakju-ro, Chuncheon-si, Gangwon-do 200-704 Republic of Korea

**Keywords:** Tuberculosis, Vitamin D, Cytokine

## Abstract

**Background:**

Vitamin D levels are associated with the extent of mycobactericidal activity. Interleukin (IL)-15 and IL-32 play roles in the vitamin D-mediated tuberculosis (TB) defense mechanism. Vitamin D induces IL-1β, which plays an important role in terms of resistance to TB. We evaluated whether the levels of vitamin D-related cytokines distinguished between those with active TB and latent TB infection (LTBI).

**Methods:**

In total, 50 TB-infected patients (25 with active TB and 25 with LTBI following a TB outbreak in a high school) were enrolled. Plasma 25-hydroxyvitamin D (25[OH]D), IL-15, IL-32, and IL-1β levels were measured via enzyme-linked immunosorbent assays. *Mycobacterium tuberculosis*-specific antigen-induced and unstimulated cytokine levels were measured in the supernatants of the QuantiFERON TB Gold-In-Tube (QFT-GIT) assay.

**Results:**

Plasma 25(OH)D and plasma IL-15 levels were lower in patients with active TB than in LTBI subjects (25(OH)D: 16.64 ng/mL vs. 21.6 ng/mL, *P* = 0.031; IL-15: 148.9 pg/mL vs. 189.8 pg/mL, *P* = 0.013). Plasma 25(OH)D levels correlated with the plasma levels of IL-15 and IL-1β in TB-infected patients. In addition, the plasma 25(OH)D levels correlated positively with the level of unstimulated IL-15 (IL-15_nil_) and negatively with that of TB antigen-stimulated IL-32 (IL-32_TB_) in QFT-GIT supernatants. Although the IL-15_nil_ and IL-15_TB_ levels were higher in LTBI subjects than patients with active TB, the IL-32_nil_ and IL-32_TB_ levels were higher in the latter patients. A combination of the IL-15_nil_ and IL-32_TB_ levels accurately predicted 91.3% of active TB patients and latent subjects, with an area under the curve of 0.964.

**Conclusions:**

Our preliminary data showed that the levels of the vitamin D-related cytokines IL-15 and IL-32 differed between active TB patients and LTBI subjects. This result might be used as a basic data for developing biomarkers distinguishing between active TB and LTBI.

## Background

Tuberculosis (TB) is the leading cause of death from a single infectious agent, ranking above Human immunodeficiency virus (HIV), causing an estimated 1.3 million deaths annually in HIV-negative subjects [1]. New diagnostics, treatments, and vaccines are required. Vitamin D plays an immunomodulatory role in several autoimmune diseases, altering T cell responses and protecting the epithelial mucosal barrier against inflammation [2, 3]. Similarly, vitamin D protects against *Mycobacterium tuberculosis* infection by modulating immune responses, stimulating the production of antimicrobial peptides such as cathelicidin and promoting autophagy [4–7]. Vitamin D regulates the production of interleukin (IL)-6, IL-17, and IL-10 in patients with congestive heart failure and inflammatory bowel disease [2, 8]. Several studies have evaluated the clinical utilities of the levels of TB antigen-stimulated serum cytokines in terms of differentiating active from latent TB [9–11]. However, few studies have explored the levels of vitamin D-related cytokines both in serum and in the supernatants of the QuantiFERON TB Gold-in-Tube (QFT-GIT) test for TB.

IL-15 plays a protective role against *M.tuberculosis* and is used as an adjuvant during vaccination against various diseases [12–14]. Krutzik et al. found that IL-15 mediated a vitamin D-dependent antimicrobial pathway [15]. IL-32 augments immunity to *M*. *tuberculosis* by increasing the expression levels of the vitamin D receptor and antimicrobial peptides, as well as caspase-mediated apoptosis or autophagy [16, 17]. 1,25-dihydroxyvitamin D3 significantly increased the levels of IL-1β associated with resistance to TB [18]. Here, we explored the associations between the levels of vitamin D and several cytokines in sera and QTF-GIT supernatants, and whether these levels distinguished those with active and latent TB infections (LTBI).

## Methods

### Study population

In 2017, a TB outbreak occurred in a high school of Chuncheon city in South Korea. The first patient diagnosed with TB in April 2017 and two additional cases were identified in September 2017. The Korea Centers for Disease Control and Prevention established 1st National TB control Plan (2013–2017) based on Public-Private Mix (PPM) TB control project and TB contact surveillance through TB patient monitoring and epidemiologic investigation [19]. It detected additional TB patients through TB contact investigation and provided checkups and treatment to latent TB infection.

An epidemiological investigation involving all students and staff was then performed in line with the national LTBI treatment policy [20]. The interferon gamma-release assay (IGRA) was performed with the aid of the QFT-GIT, and chest X-rays were taken. The QFT-GIT results were scored as negative, indeterminate, or positive (cutoff 0.35 IU/mL), as recommended by the manufacturer. A total of 106 subjects yielding positive results on the QFT-GIT test or with abnormal chest X-rays were transferred to Chuncheon Sacred Heart Hospital or Kangwon National University Hospital.

High-resolution chest tomography (HRCT) was performed on all subjects; HRCT distinguishes active TB from LTBI [21]. Those positive on the QFT-GIT test and lacking active TB lesions on HRCT were diagnosed with LTBI. Active TB was diagnosed if sputum specimens were culture-positive for *M*. *tuberculosis* or if the clinical TB criteria were met [22]. These included typical cavities and branching centrilobular nodules evident on HRCT, and good clinical and radiographic responses to anti-TB treatment.

**Fig. 1:**
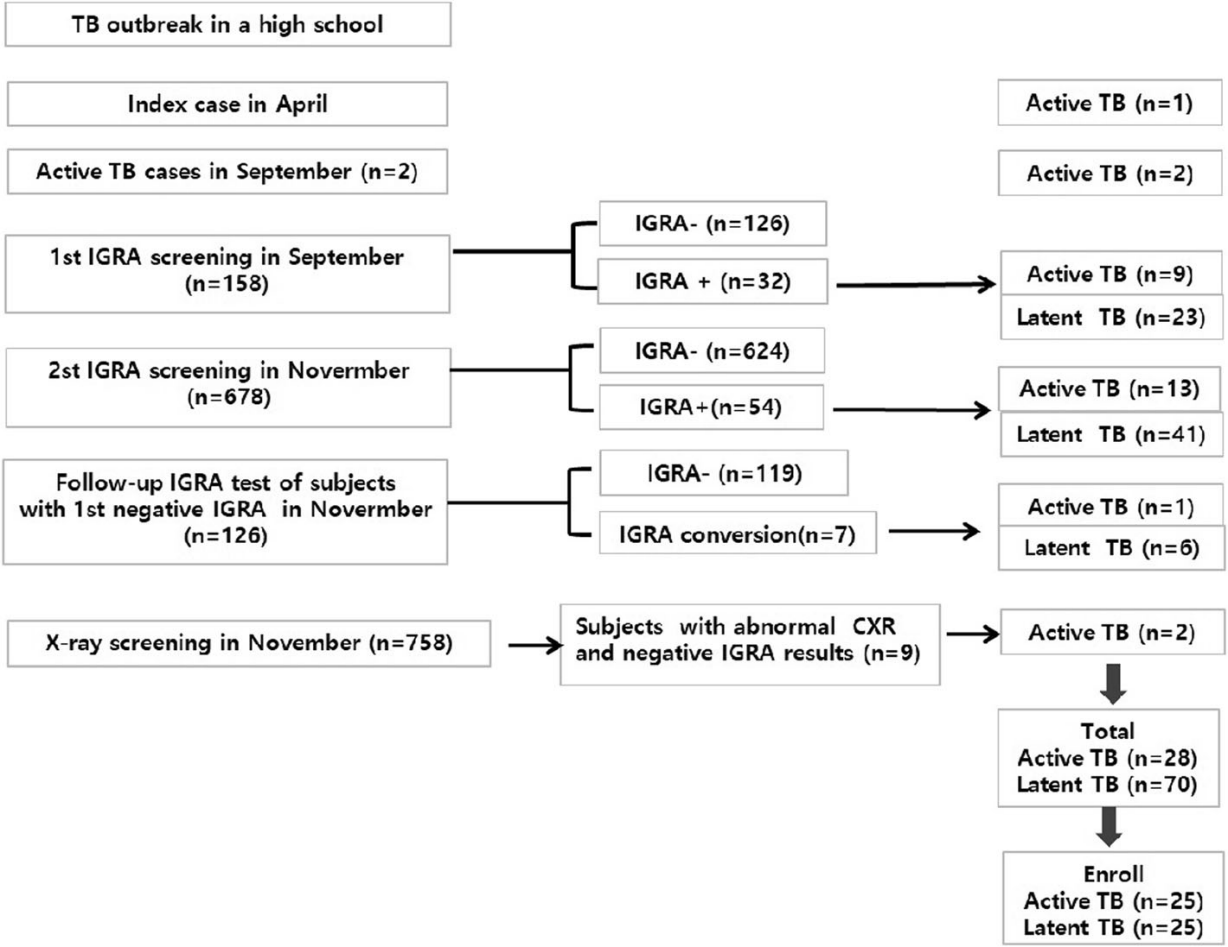
Schema of the TB outbreak investigation and management

We commenced anti-TB medication for active TB patients; LTBI subjects were treated as dictated by the national TB control plan. In total, 28 TB patients and 70 LTBI subjects were diagnosed. Finally, 25 patients with active TB and 25 LTBI subjects gave written informed consent for inclusion in the present study. The consent of the parents was obtained because the age of participants was under 19 years old (Fig. 1).

### Specimen collection and QFT-GIT assay

Blood samples were voluntarily given at enrolment, before therapy with anti-tuberculous drugs. The QFT-GIT assay was performed according to the manufacturer’s instructions (Qiagen, Hilden, Germany); we evaluated both active TB patients and LTBI subjects. After incubation for 20 h at 30 °C with *M*. *tuberculosis*-specific antigens (ESAT-6, CFP-10, and TB 7.7), plasma supernatants were collected after centrifugation and stored at − 80 °C prior to the assay.

### Measurement of vitamin D and cytokine levels by ELISA

Plasma 25-hydroxyvitamin D levels [25(OH) D levels] were measured with the aid of a commercial enzyme-linked immunosorbent assay (ELISA) kit (Mybiosource, San Diego, CA, USA) according to the manufacturer’s instructions. The plasma levels of IL-15, IL-32, and IL-1β in all patients were measured using commercial ELISA kits according to the manufacturers’ instructions (IL-15: Raybiotech, Norcross, GA, USA, Catalog # ELH-IL15; IL-32: Mybiosource, Catalog # MBS720152; and IL-1β: Cusabio, Houston, TX, USA, Catalog # CSB-E08053h); the levels of these materials in QFT-GIT supernatants from the QuantiFERON nil and antigen tubes were also measured.

### Statistical analysis

Data are expressed as medians with interquartile ranges (IQRs); we performed nonparametric analysis. Continuous variables were compared using the Kruskal–Wallis test or the Mann–Whitney U-test. Correlations between vitamin D and cytokine levels were sought using Spearman’s rank test. The most appropriate cutoff values for vitamin D and cytokine levels were derived by reference to the maximal Youden indices of receiver operating characteristic (ROC) curves. Multivariate logistic regression analysis was used to determine the probability of active TB in each patient based on an equation featuring independent factors. Statistical analysis was performed with the aid of GraphPad Prism (GraphPad, San Diego, CA, USA) and the Statistical Package for the Social Sciences (v. 20; SPSS, Chicago, IL, USA).

## Results

### Study participants

We enrolled 25 patients with active pulmonary TB and 25 LTBI subjects. The demographic characteristics of all participants are shown in Table 1. The age and gender distributions of the active TB and LTBI groups did not differ. Two patients in the active TB group yielded negative IGRA results, and 15 patients in that group were acid-fast bacillus (AFB) culture-negative. As TB was diagnosed soon after contact, lung involvement was limited to one lobe in most cases.

**Table 1:** Basic characteristics of the study population

	Active TB (*n* = 25)	LTBI (*n* = 25)
Age†	18 (17–18)	18 (17–18)
Male, n (%)	25 (100)	25 (100)
BMI†	21.8 (19.7–24.2)	23.2 (21.5–26.1)
BCG vaccination history, n (%)	25 (100)	25 (100)
Pulmonary TB diagnosis, n (%)		
Positive AFB smear	1 (4%)	
Positive AFB culture	10 (20%)	
Extent of lesion evident on HRCT		
Single lobe involvement	20 (80%)	
Multiple lobe involvement	5 (20%)	
Positive on IGRA test, n (%)	23 (92%)	25 (100%)
IFNγ_TB-nil_ of QFT-GIT†	4.93 (1.78–10)	0.92 (0.64–2.36)

†Age in years; body mass index (BMI) in kg/m^2^

### Levels of plasma cytokines, and M. Tuberculosis antigen-induced and unstimulated cytokines

**Fig. 2:**
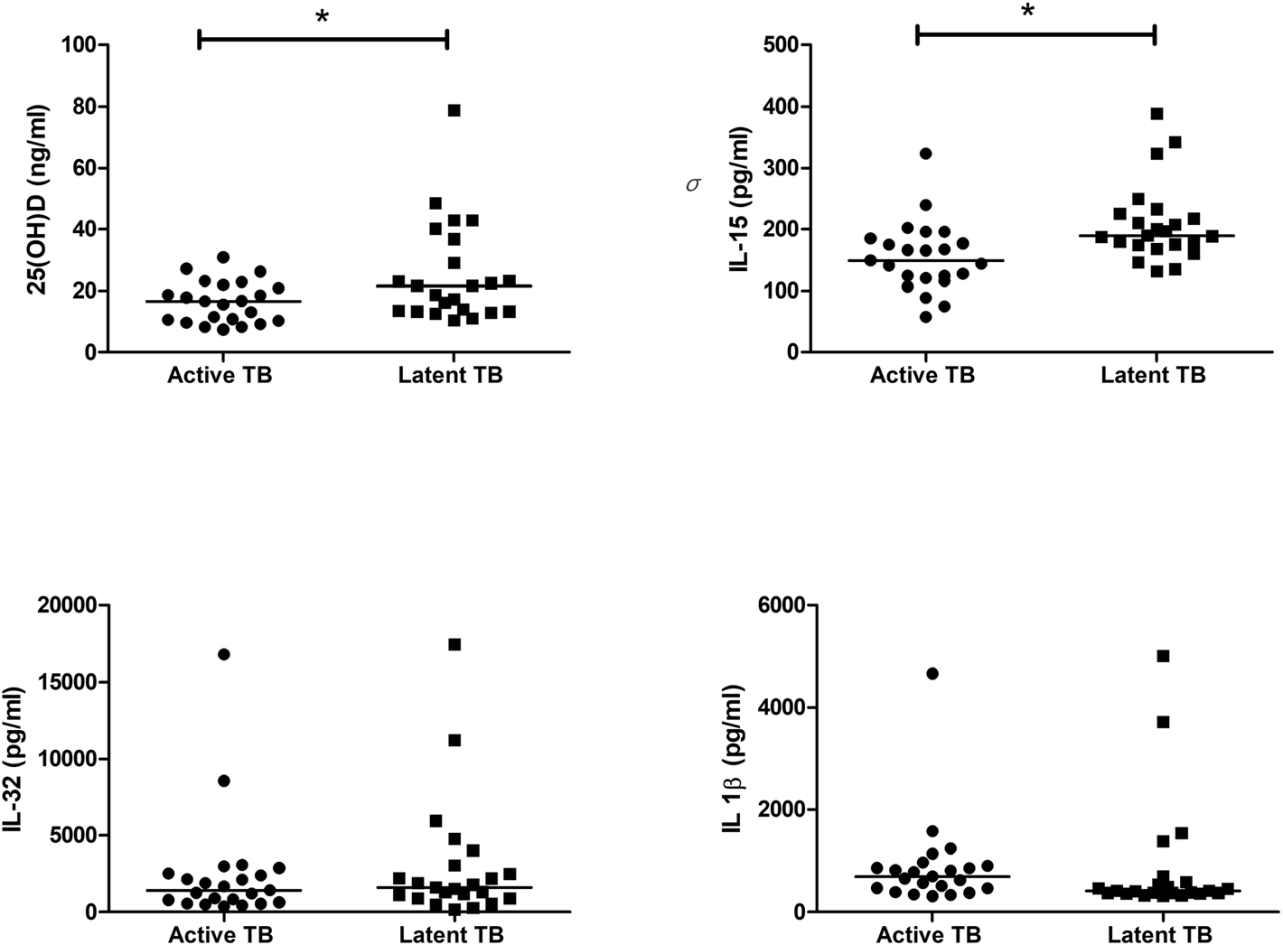
Plasma (**a**) 25(OH)D, (**b**) IL-15, (**c**) IL-32, and (**d**) IL-1β levels in the active TB group and LTBI subjects. The horizontal lines indicate median values. 25(OH)D: 25-hydroxyvitamin D

As shown in Fig. 2, the 25(OH)D level differed between those with active TB (median 16.64 IU/mL, IQR: 13.32–36.81) and latent TB (median 21.60 IU/mL, IQR: 13.32–36.81 IU/mL, *P* = 0.033). The plasma IL-15 was significantly lower in patients with active TB (median 148.9 pg/mL, IQR 120.6–184.8 pg/mL) than LTBI subjects (median 189.8 pg/mL, IQR 174.2–225.7 pg/mL). However, the plasma IL-32 levels and plasma IL-1β level did not differ between active TB and LTBI subjects.

**Fig. 3:**
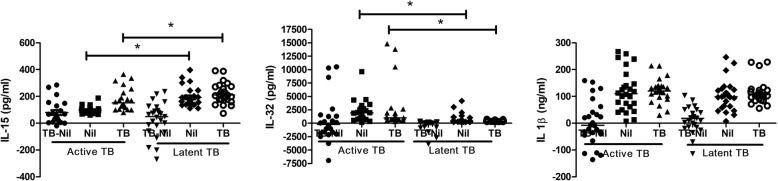
IL-15, IL-32, and IL-1β levels measured via the QFT-GIT assay in those with active TB and LTBI. TB-nil: M. tuberculosis antigen-dependent response as measured by the difference in cytokine levels between the nil and TB antigen-coated tubes. The horizontal lines indicate median values

Figure 3 shows the IL-15, IL-32, and IL-1β levels following antigen stimulation during the QFT-GIT test in the active TB and LTBI groups. The median levels of unstimulated IL-15 (IL-15_nil_) and the IL-15 response to TB antigens (IL-15_TB_) were significantly lower in those with active TB than the LTBI group (IL-15_nil_: active TB 94.62 pg/mL, IQR 79.08–109.6 pg/mL; LTBI 162.8 pg/mL, IQR 146.1–222.6 pg/mL, *P* < 0.001; IL-15_TB_: active TB 152.2 pg/mL, IQR 114.7–234.6 pg/mL, LTBI 210.4 pg/mL, IQR 177.4–272.4 pg/mL, *P* = 0.025). On the contrary, the medians of the unstimulated IL-32 (IL-32_nil_) and IL-32 responses to TB antigen (IL-32_TB_) were significantly higher in the active TB than the LTBI group (IL-32_nil_: active TB 1925 pg/mL, IQR 766.5–2927 pg/mL; LTBI 410.2 pg/mL, IQR 306.7–842.9 pg/mL, *P* < 0.001; IL-32_TB_: active TB 992.8 pg/mL, IQR 741.4–2132 pg/mL; LTBI 405.2 pg/mL, IQR 361.3–552.0 pg/mL, *P* < 0.001). Neither the unstimulated IL-1β level nor the IL-1β response to TB antigen differed between the active TB and LTBI groups.

### Correlation between cytokine and vitamin D levels

**Fig. 4:**
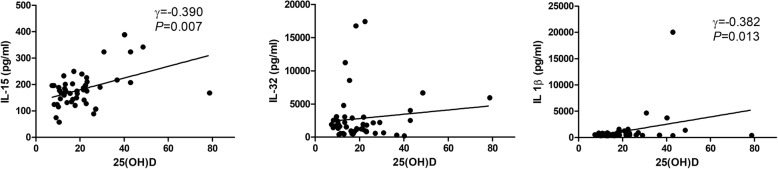
Correlations between plasma 25(OH)D and cytokine levels in the active and latent TB groups.25(OH)D: 25-hydroxyvitamin D

**Fig. 5:**
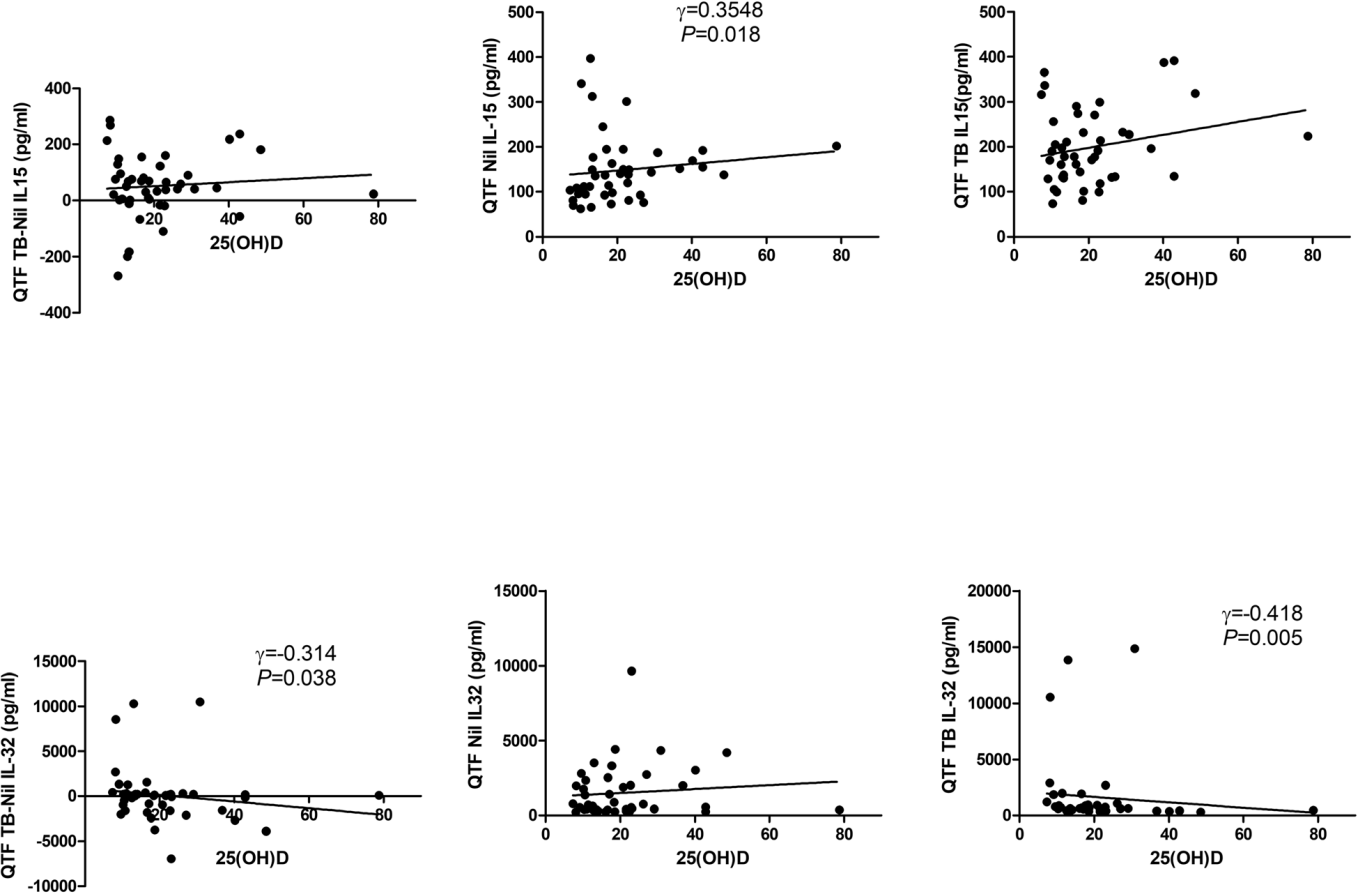
Correlations between plasma 25(OH)D levels and cytokine levels in the QFT supernatants of the active and latent TB groups. 25(OH)D: 25-hydroxyvitamin D

In TB-infected patients (25 active TB and 25 LTBI), the 25(OH)D level correlated positively with those of plasma IL-15 (*r* = 0.390, *P* = 0.007) and plasma IL-1β (*r* = 0.382, *P* = 0.013) (Fig. 4). In terms of the QFT-GIT test data, 25(OH)D levels correlated positively with the IL-15_nil_ (*r* = 0.355, *P* = 0.018) and negatively with the IL-32_TB_ (r = − 0.314, *P* = 0.038) and IL-32_TB–nil_ (*r* = − 0.418, *P* = 0.005) values (Fig. 5). We found no significant correlation between the plasma concentrations of cytokines. Significant correlations were evident between the IL-15_nil_ value and those of other cytokines measured in QFT-GIT supernatants_._ The IL-15_nil_ value correlated negatively with the IL-32_nil_ (*r* = − 0.314, *P* = 0.038), IL-32_TB_ (*r* = − 0.314, *P* = 0.038), and IL-1β_nil_ (*r* = − 0.314, *P* = 0.038) values.

### Capacity of vitamin D/cytokine levels to discriminate between active and latent TB

To evaluate the use of vitamin D-related cytokine levels for the diagnosis of active TB and LTBI, we performed ROC analysis of several markers in plasma and supernatants of the QFT test (Table 2). IL-15_nil_ exhibited the highest area under the curve (AUC) (0.951), followed by IL-32_TB_ (0.941), IL-32_nil_ (0.811), and plasma IL-15 (0.777). At a cut-off of 167.6 pg/mL, plasma IL-15 afforded 65.2% sensitivity and 82.6% specificity, whereas IL-15_nil_, at a cut-off of 137.2 pg/mL, afforded a sensitivity and specificity of 90.9 and 88% respectively. At a cut-off of 174.2 pg/mL, IL-15_TB_ afforded 63.6% sensitivity and 80.0% specificity. IL-32_nil_ afforded 86.4% sensitivity and 76% specificity at a cut-off of 662.3 pg/mL and IL-32_TB_ 90.9% sensitivity and 92.0% specificity at a cut-off of 627.6 pg/mL. We used multivariate logistic regression to analyze the cytokines exhibiting the highest AUCs (IL-15_nil_ and IL-32_TB_). Both were independent predictors of active TB, as revealed by the equation:

**Table 2:** Performance of different analytes used to discriminate between latent and active tuberculosis

Biomarker	AUC	Sensitivity	Specificity	PPV	NPV	LR+	LR-
Plasma IL-15 < 167.6 pg/mL	0.7769	65.2%	82.6%	78.9	70.3	3.75	0.42
IL-15_nil_ < 137.2 pg/mL	0.9509	90.9%	88.0%	88.3	90.6	7.58	0.1
IL-15_TB_ < 174.2 pg/mL	0.6927	63.6%	80.0%	76.1	68.7	3.18	0.45
IL-32_nil_ ≥ 662.3 pg/mL	0.8109	86.4%	76.0%	78.3	84.8	3.6	0.18
IL-32_TB_ ≥ 627.6 pg/mL	0.9491	90.9%	92.0%	91.8	90.9	11.36	0.1
Vitamin D < 12.05 ng/mL	0.6843	39.1%	91.3%	81.8	60	4.49	0.67
IL-15_nil_ < 137.2 pg/mL or IL-32_TB_ ≥ 627.6 pg/mL	0.964	100%	82.6%	85.2	100	5.75	0

ROC curves were drawn

ROC: receiver operating characteristic; AUC: area under the curve

PPV, positive predictive value; NPV, negative predictive value; LR+, likelihood ratio-positive; LR−, likelihood ratio-negative

Logit (probability) = Ln (probability/1 – probability) = [3.357 × (IL-15_nil_ < 137.2 pg/mL)] + [3.890 × (IL-32_TB_ ≥ 627.6 pg/mL)] – 3.785.

ROC analysis showed that the predicted probability had an AUC of 0.964 at an optimal cut-off of 0.208 (100% sensitivity and 82.6% specificity). Active TB was favored if one of two criteria (IL-15_nil_ < 137.2 pg/mL or IL-32 _TB_ ≥ 627.6 pg/mL) was met. This two-factor model afforded increased sensitivity at the cost of mildly reduced specificity compared to models involving other biomarkers. There was no false-negative patient in the active TB group but three false-positive patients in the LTBI group. Notably, in two patients, active TB was correctly diagnosed based on both criteria, although the initial IGRA results were negative.

## Discussion

Several studies have described associations between TB and vitamin D deficiency [23–26]. One epidemiological study found that Korean TB patients exhibited a higher prevalence of vitamin D deficiency than healthy subjects [23]. Arnedo-Pena et al. found that vitamin D concentration correlated with a low incidence of conversion after TB infection [27]. Similarly, Balcells et al. found that the most important determinant of vitamin D deficiency was seasonality and LTBI acquisition risk was higher in spring compared with other season of the year [28]. Our results are consistent with those of a Spanish study demonstrating that a severe deficit of vitamin D (< 10 ng/mL) could be used to distinguish active TB from LTBI [29].

Several in vitro studies found associations between vitamin D levels and antimicrobial cytokine release. IL-15 mediates TLR2/1-induced induction of the CYP27b1, VDR, and cathelicidin as vitamin D-dependent pathways involved in macrophage differentiation [15]. Montaya et al. found that IL-32 induced vitamin D-dependent antimicrobial peptides during the IL-15-mediated defense response of the macrophage gene network [17]. Vitamin D triggered IL-1β secretion in macrophages of TB patients, effectively restricting bacterial growth [18]. 1,25-dihydroxyvitamin D-induced IL-1β secretion from macrophages, and reduced the mycobacterial burden by modulating paracrine signaling [30].

We showed that there is a significant difference in vitamin D-related cytokines between active TB patients and latent TB subjects. The plasma IL-15 and IL-15_nil_ levels correlated positively with the vitamin D level. The plasma IL-15 levels and IL-15_nil_ and IL-15_TB_ levels were significantly higher in those with LTBI than active TB, but the TB-specific antigen-dependent IL-15 level (IL-15_TB–nil_) was not. This result is similar to that of Chandrashekara et al., who found that the serum IL-15 level was significantly lower in a TB-infected group than healthy controls [31]. IL-15 boosts the immune response to infection by activating NK cells and enhancing priming of CD4/CD8 cells in terms of IFN–r production in patients with TB [32, 33]. Frahm et al. reported that the IL-15_TB–nil_ level was significantly higher in those with active than latent TB [34]. This differs from our finding, perhaps because the cited study included those with HIV infections and patients who had completed TB treatment at the time of enrolment.

The plasma IL-32 level did not distinguish active from latent TB and did not correlate with the vitamin D level, but the IL-32_TB_ level was significantly higher in those with active than latent TB, and correlated negatively with the vitamin D level in the present study. To the best of our knowledge, this is the first study to measure IL-32 levels in QFT-GIT supernatants.

Previous studies yielded inconsistent results. Bao et al. reported that the serum IL-32 level of a TB group was significantly higher than that of a control group [35] and later reported that lung IL-32 expression was increased in patients with active TB [36]. On the contrary, Montaya reported that the IL-32 mRNA level in peripheral blood was lowest in those with active TB, compared to those with latent TB and healthy controls [17]. Both IL-15 and IL-32 protect against TB [13, 37]. Weighted gene co-expression network analysis revealed that IL-32 was linked to IL-15 in terms of induction of the defense response network [17]. However, in our present in vivo study, we found differences in the expression levels of the two cytokines in those with active TB and LTBI subjects. The reason may be that IL-32 production could be affected by other signal pathways such as IL-18 and caspase-1. Further research is needed to conclude the role of IL-32 according to the spectrum of TB infection.

We found that the plasma IL-1β level correlated positively with the vitamin D level but did not distinguish active TB from LTBI. Wergeland et al. showed that the IL-1β level of unstimulated (“nil”) supernatants was lower in an active TB than an LTBI group [38], but we found that IL-1β levels in QFT-GIT supernatants did not differ between the active TB and LTBI groups.

We chose optimal cytokine cutoff levels distinguishing active TB from LTBI. We performed logistic regression by reference to vitamin D and vitamin D-related cytokine levels. On multivariate logistic regression, an IL-15_nil_ level < 137.2 pg/mL and IL-32_TB_ level ≥ 627.6 pg/mL independently predicted active TB. When the probability predicted by the regression model was ≥0.208 (100% sensitivity and 82.6% specificity), active TB was favored when QFT-GIT analysis fulfilled one of above two criteria (IL-15_nil_ and IL-32_TB_). The predictive factor model combining IL-15_nil_ and IL-32_TB_ improved the sensitivity compared with each marker and continued high sensitivity.

Our study had several limitations. First, there is no gold standard test for latent TB infection, LTBI was defined by QFT-GIT to assess the immunologic reaction to specific antigens of M. tuberculosis in this study. Second, we lacked healthy controls of TB outbreak in a high school. Third, validation is needed in larger population because the sample size is small and potential for type I error exists. Because this study is done through TB contact investigation after TB outbreak, the additional subjects could not be recruited.

The strength of the study is that the LTBI and active TB groups were enrolled from a TB outbreak in a high school. Therefore, age, gender, and the TB exposure environment were near-identical in the two groups.

## Conclusions

In summary, the levels of vitamin D, plasma IL-15, IL-15_nil_, and IL-32_TB_ differentiated active TB from LTBI. The correlations between vitamin D and cytokine levels suggest that certain cytokines mediate the TB-protective effects of vitamin D. Measurement of both IL-15_nil_ and IL-32_TB_ in QFT-GIT supernatants accurately discriminated active TB from LTBI. Further studies in more diverse populations are required to validate the utility of these markers.

## Abbreviations

25(OH)D: 25-hydroxyvitamin D
AFB: acid-fast bacillus
AUC: Area under the curve
ELISA: Enzyme-linked immunosorbent assay
HIV: Human immunodeficiency virus
HRCT: High-resolution chest tomography
IL: Interleukin
IQR: interquartile ranges
LTBI: Latent tuberculosis infection
QFT-GIT: QuantiFERON TB Gold-In-Tube
ROC: Receiver operating characteristic
TB: Tuberculosis
